# Molecular Analysis of *RNF213* Gene for Moyamoya Disease in the Chinese Han Population

**DOI:** 10.1371/journal.pone.0048179

**Published:** 2012-10-23

**Authors:** Zhiyuan Wu, Hanqiang Jiang, Lei Zhang, Xiao Xu, Xinju Zhang, Zhihua Kang, Donglei Song, Jin Zhang, Ming Guan, Yuxiang Gu

**Affiliations:** 1 Department of Laboratory Medicine, Huashan Hospital, Shanghai Medical College, Fudan University, Shanghai, China; 2 Department of Neurosurgery, Huashan Hospital, Shanghai Medical College, Fudan University, Shanghai, China; 3 Department of Equipment, Huashan Hospital, Shanghai Medical College, Fudan University, Shanghai, China; 4 Central Laboratory, Huashan Hospital, Shanghai Medical College, Fudan University, Shanghai, China; 5 Department of Nursing, Huashan Hospital, Shanghai Medical College, Fudan University, Shanghai, China; Oslo University Hospital, Norway

## Abstract

**Background:**

Moyamoya disease (MMD) is an uncommon cerebrovascular disorder characterized by progressive occlusion of the internal carotid artery causing cerebral ischemia and hemorrhage. Genetic factors in the etiology and pathogenesis of MMD are being increasingly recognized. Previous studies have shown that the *RNF213* gene was related to MMD susceptibility in the Japanese population. However, there is no large scale study of the association between this gene and MMD in the Chinese Han population. Thus we designed this case-control study to validate the R4810K mutation and to define the further spectrum of *RNF213* mutations in Han Chinese.

**Methodology/Principal Findings:**

Genotyping of the R4810K mutation in the *RNF213* gene was performed in 170 MMD cases and 507 controls from a Chinese Han population. The R4810K mutation was identified in 22 of 170 MMD cases (13%), including 21 heterozygotes and a single familial homozygote. Two of the 507 controls (0.4%) were heterozygous R4810K carriers. The R4810K mutation greatly increased the risk for MMD (OR = 36.7, 95% CI: 8.6∼156.6, *P* = 6.1 E-15). The allele frequency of R4810K was significantly different between patients with ischemia and hemorrhage (OR = 5.4, 95% CI: 1.8∼16.1, *P* = 0.001). Genomic sequencing covering *RNF213* exon 40 to exon 68 also identified eight other non-R4810K variants; P4007R, Q4367L, A4399T, T4586P, L4631V, E4950D, A5021V and M5136I. Among them A4399T polymorphism was found in 28/170 cases (16.5%) and 45/507 controls (8.9%) and was associated with MMD (OR = 2.0, 95% CI: 1.2∼3.3, *P* = 0.004), especially with hemorrhage (OR = 2.8, 95% CI: 1.2∼6.5, *P* = 0.014).

**Conclusions:**

*RNF213* mutations are associated with MMD susceptibility in Han Chinese. The ischemic type MMD is particularly related to the R4810K mutation. However, A4399T is also a susceptible variant for MMD, primarily associated with hemorrhage. Identification of novel variants in the *RNF213* gene further highlights the genetic heterogeneity of MMD.

## Introduction

Moyamoya disease (MMD) is an uncommon cerebrovascular disease characterized by stenosis and occlusion of terminal segments of the bilateral internal carotid arteries with abnormal net-like vessels at the base of the brain [1]. MMD has many clinical manifestations, such as transient ischemic attacks (TIAs), cerebral infarction, seizure, headache, and intracranial hemorrhage [2]. The most common signs in clinical practice are still cerebral ischemia and intracranial hemorrhage which often have serious consequences [3]. However, the etiology of this condition is still unclear.

Epidemiologically, MMD is more prevalent in East Asia, especially in Japan and South Korea, than in western countries [4]–[7]. Although nationwide epidemiological data on MMD in China is currently poorly stated, an epidemiological survey in Nanjing, China from 2007 to 2009 found a prevalence of 0.43 cases/100,000 annually. The incidences of ischemic type and hemorrhagic type were 0.16 cases/100,000 and 0.22 cases/100,000 people per year, respectively [8]. Familial MMD (FMMD) individuals accounted for 10–15% cases [9]. The incidence of MMD in monozygotic twins was as high as 80% [10]. MMD often occurred alongside hereditary diseases, such as neurofibromatosis type I and Down's syndrome. These phenomena imply that genetic factors may play a potential important role in the pathogenesis of the disease.

Genetic studies on MMD using linkage analysis have discovered five candidate loci: chromosome 3p24–26, 6q25, 8q23, 12p12 and 17q25 [11]–[14]. Genome-wide association studies using single nucleotide polymorphisms (SNPs) also revealed several susceptibility genes for MMD: *ACTA2*, *RPTOR*, *PDGFRB* and *TGFB1* [15]–[17]. However, none of the results have been replicated yet. Mineharu et al. validated genetic variants in the unique locus of 17q25.3 as a main risk factor of familial MMD [18]. Kamada et al. recently identified *RNF213* as a MMD susceptibility related gene in a genome-wide, locus-specific association study [19]. Further large scale sequencing analysis from Liu et al. confirmed R4810K (rs112735431) as the only genetic variant common in 42 families and discovered other non-R4810K mutations in *RNF213* in East Asian cases and in Caucasian cases [20]. However, so far, there is no large scale case control confirmation of the association between this locus and MMD prevalence in Chinese Han population. In order to determine whether *RNF213* does also increase the susceptibility to MMD in the Han Chinese, we performed molecular analysis of 170 consecutive cases with MMD including 5 familial ones.

**Table 1:** Basic characteristics of patients with MMD and normal controls.

	Patients with MMD	controls
Subjects	170	507
Male	96 (56.5%)	130 (25.6%)
Age in years	35.8±13.2	37.2±16.9
Age group		
<18 years	21 (12.4%)	7 (1.4%)
≥18 years	149 (87.6%)	500 (98.6%)
Symptoms at onset		
Ischemia	84 (49.4%)	0 (0%)
Hemorrhage	86 (50.6%)	0 (0%)
Familial MMD	5 (2.9%)	0 (0%)

## Materials and Methods

### Patients

The study involves 170 patients with MMD at Huashan Hospital, Fudan University (Shanghai, China) collected from January 2007 to December 2011. Subjects included 96 men and 74 women. All except 5 patients were individuals without any family history of MMD. The control group consisted of 507 healthy adults from the routine laboratory tests. Basic characteristics of patients were list in Table 1. All subjects were Han Chinese. In compliance with the Helsinki Declaration of 1975 as revised in 1996, this study was approved by the Institutional Review Board of the Huashan Hospital. All the participants provided written informed consent for the sample collection and subsequent analysis.

**Figure 1:**
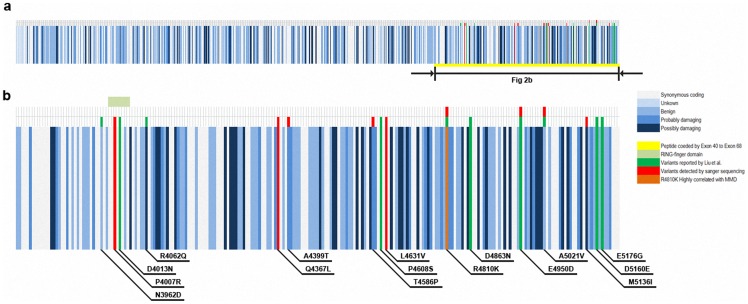
Genetic variants heat map of RNF213 generated from 1000 genomes database. (a) variants in whole genome of RNF213. (b) The 35kb highly variable region flanking RNF213 R4810K mutation. The intensity of blue bars indicates the predicted risk of each variation.

### Genotyping of *RNF213* R4810K with unlabeled probe high resolution melting analysis

We investigated the association between *RNF213* R4810K mutation and the risk of MMD using high resolution melting analysis (HRMA) with an unlabeled probe. Genomic DNA isolated from peripheral blood lymphocytes was extracted with a QIAamp DNA Blood Kit (Qiagen, Valencia, CA). All oligonucleotide primers and probes were obtained from Sango Biotech Co. (Shanghai, China). The primers were designed as follows: RNF213-4810F: 5′-aagcagttccagaacgtcca-3′; RNF213-4810R: 5′-agtcctggtcctgtcagagc-3′. The probe was an oligonucleotide blocked at the 3′end and the sequence was as follows: 5′-tgaatacagctccatcagaggcttcctcagc-3′. The target product was 105 bp in length.

**Figure 2:**
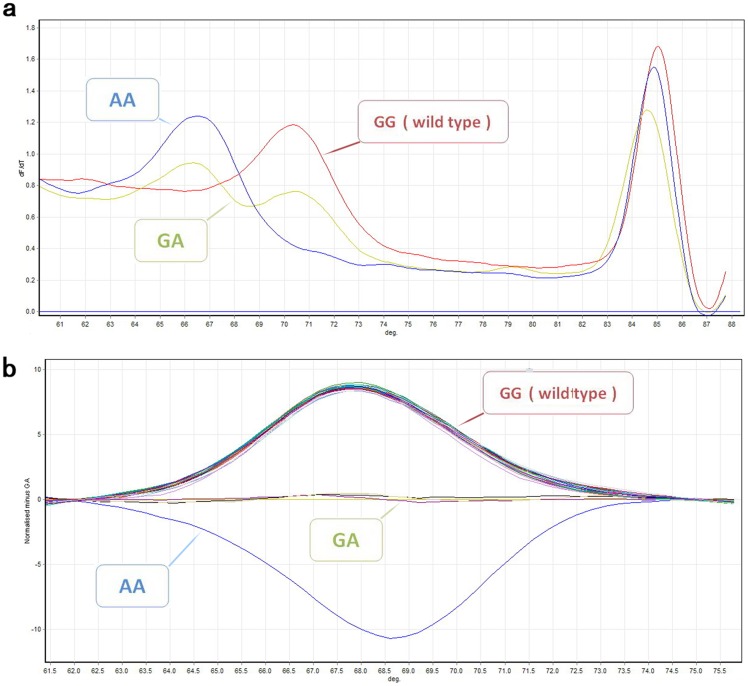
High resolution melting analysis for genotyping of RNF213 R4810K. (a) Derivative melting curves of unlabeled probes and amplicon. Three genotypes (AA, AG, and GG) were discriminated as indicated in the probe region. (b) Difference curves obtained by subtracting each curve from one heterozygote (AG) curve. Three genotypes (AA, AG, and GG) are shown.

Genotyping of the *RNF213* R4810K mutation was carried out by unlabeled probe HRMA. Unlabeled probe HRMA was developed from asymmetric PCR. After asymmetric PCR, a large number of superfluous single strands are hybridized with unlabeled probes at the lower temperature. With the increasing temperature in the melting curve analysis, it produces two types of melting curves. The part of curve corresponding with low melting temperatures represents the melting region of probe and single strand product.

**Figure 3:**
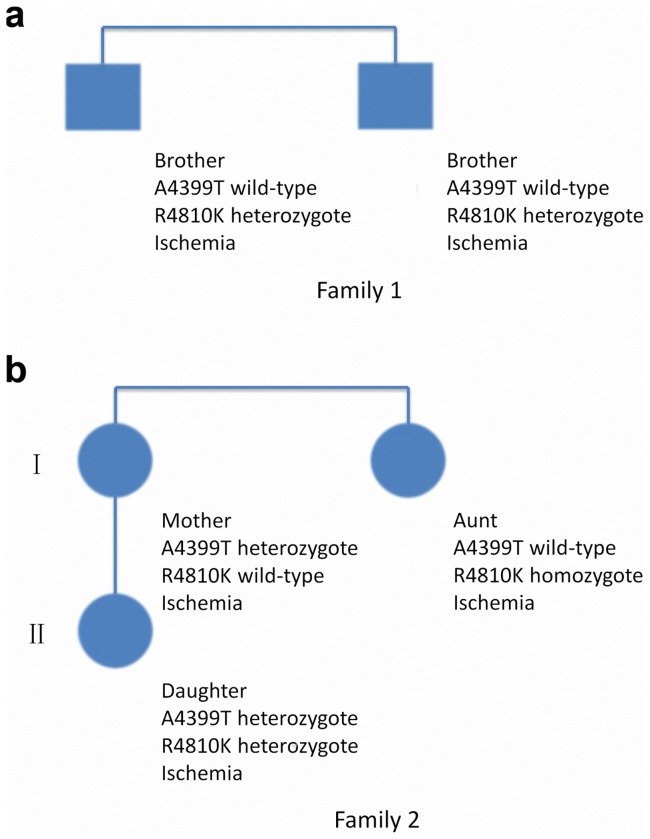
Genetic background of the five familial cases. (a) Family 1, two brothers both harboring a heterozygous R4810K mutation. (b) Family 2, mother and aunt are whole sisters with A4399T heterozygote and R4810K homozygote respectively. The daughter has both the heterozygous A4399T and R4810K variants. All the patients were diagnosed with the ischemia subtype.

**Table 2:** Genotype and allele distribution of RNF213 R4810K in MMD.

		Genotype frequency, no.(%)				Allele frequency,no.(%)			OR(95%CI)
SNP, population	No. of subjects	Minor homozygote	Heterozygote	Major homozygote	P value	Minor allele	Major allele	P value	
Genotype or allele		A/A	A/G	G/G		A	G		
Cases	170	1 (0.6)	21 (12.4)	148 (87.0)	2.16E-13	23 (6.8)	317 (93.2)	6.11E-15	36.71(8.61–156.58)
Controls	507	0 (0.0)	2 (0.4)	505 (99.6)		2 (0.2)	1012(99.8)		

Asymmetric PCR was performed on a Rotor-Gene Q (Qiagen) in 20 μl of reaction volume. The master mix contained: 1×reaction buffer (TaKaRa BIO, Shiga, Japan), 1×SYTO9 (Invitrogen, Carlsbad, CA), 200 μM dNTP, 0.05 μM forward-primer, 0.5 μM reverse-primer, 0.5 μM probe, 2 mM MgCl_2_, 1 U TaKaRa Ex Taq DNA polymerase (TaKaRa BIO), and 1 μl (15–25 ng/μL) of genomic DNA. All amplifications began with a 5 minute hold at 95°C followed by 50 cycles of denaturation at 95°C for 15 seconds, annealing at 58°C for 30 seconds, and an extension at 72°C for 30 seconds.

**Table 3:** Association between RNF213 R4810K with clinical characteristics in MMD patients.

		Genotype frequency, no. (%)				Allele frequency, no. (%)			
SNP, population	No. of subjects	Minor homozygote	Heterozygote	Major homozygote	P value	Minor allele	Major allele	P value	OR (95%CI)
Genotype or allele		A/A	A/G	G/G		A	G		
Total cases	170	1	21	148		23	317		
Age group									
<18years	21	0	6	15	0.052	6	36	0.038	2.75(1.02–7.44)
≥18years	149	1	15	133		17	281		
Gender									
Female	74	1	11	62	0.345	13	135	0.193	1.75(0.75–4.12)
Male	96	0	10	86		10	182		
Symptoms at onset									
Ischemia	84	1	17	66	0.005	19	149	0.001	5.36(1.78–16.10)
Hemorrhage	86	0	4	82		4	168		
Symptoms at onset & ≥18 years (149)									
Ischemia	64	1	11	52	0.020	13	115	0.004	4.69(1.49–14.75)
Hemorrhage	85	0		81		4	166		

The PCR products were subjected to denaturation at 98°C for 2 minutes followed by cooling down to 40°C for 2 minutes to facilitate the heteroduplex formation, and then heated slowly at 0.2°C/s from 50°C to 95°C. HRMA was performed using Rotor-Gene Q 1.7 software. Positive controls were included with each assay (homozygous wild-type, heterozygous, and homozygous variants).

**Figure 4:**
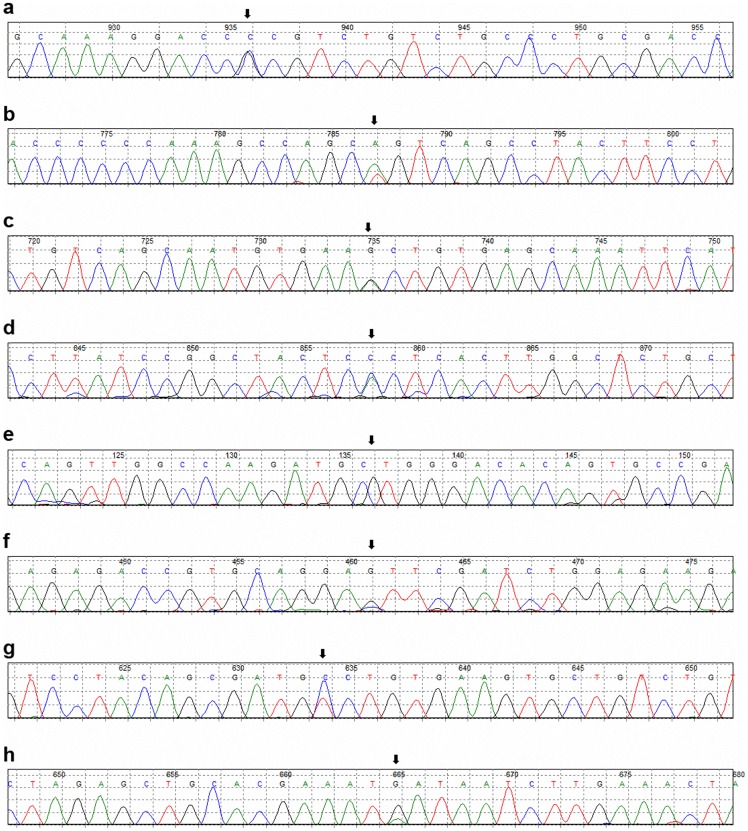
Novel mutations found by genomic sequencing of RNF213. Results from direct sequencing analyzed by Mutation Surveyor 4.06. Mutations are indicated by arrow. (a) g.107149C>G (p.P4007R). (b) g.114926A>T (p.Q4367L). (c) g.115456G>A (p.A4399T). (d) g.120087A>C (p.T4586P). (e) g.120781C>G (p.L4631V). (f) g.125960G>C (p.E4950D). (g) g.128375C>T (p.A5021V). (h) g.129275G>A (p.M5136I).

**Table 4:** Genetic variations detected in 9 MMD patients.

ID	P4007R (L)	Q4367L (L-H)	A4399T (H)	T4586P (H)	L4631V (H)	R4810K (H)	E4950D (E*)	A5021V (H*)	M5136I (H)	Family History	Phenotype
1	+					+				Familial	Ischemia
2			+							Sporadic	Hemorrhage
3			+			+				Familial	Ischemia
4			+							Familial	Ischemia
5			+			+			+	Sporadic	Ischemia
6								+		Sporadic	Ischemia
7		+		+		+				Sporadic	Ischemia
8					+					Sporadic	Ischemia
9							+			Sporadic	Hemorrhage

L = located in loop, H = located in helix, E = located in sheet, L-H = Crossing loop and helix, * = identified but with a low reliability score.

ID1: brother from family 1, ID 3: daughter from family 2, ID 4: mother from family 2.

### *RNF213* R4810K sequencing verification for HRMA results

For verification, we re-amplified 20 samples with different genotypes of each SNP as determined by unlabeled probe melting. We used the same primers that were used for sequencing analysis. Amplicons were gel purified using a QIAquick Gel Purification Kit (Qiagen). DNA sequencing analysis was performed on an ABI PRISM 310 genetic analyzer (Applied Biosystems, Foster City, CA, USA.).

**Table 5:** Genotype and allele distribution of RNF213 A4399T in MMD.

		Genotype frequency, no.(%)				Allele frequency,no.(%)			OR(95%CI)
SNP, population	No. of subjects	Minor homozygote	Heterozygote	Major homozygote	P value	Minor allele	Major allele	P value	
Genotype or allele		A/A	A/G	G/G		A	G		
Cases	170	1 (0.6)	27 (15.9)	142 (83.5)	0.008	29 (8.5)	311 (91.5)	0.004	2.01(1.24–3.26)
Controls	507	0 (0.0)	45 (8.9)	462 (91.1)		45 (4.4)	969(95.6)		

### Genomic direct sequencing and analysis of RNF213 exon 40 to exon 68

To search for other causative variants, we generated a genetic variation heat map of *RNF213* from the 1000 genomes database (http://browser.1000genomes.org/Homo_sapiens/Transcript/ProtVariations?db=coreg=ENSG00000173821r=17:78234665-78370078t=ENST00000508628) and sequenced the 35-kb genomic hypervariable region of *RNF213* from exon 40 to exon 68 (Figure 1). Genomic DNA was extracted from peripheral blood in 20 randomly selected MMD cases including three familial ones using the QiAamp DNA Blood Midi Kit (Qiagen, Hilden, Germany). PCR primers were designed by Primer 3.0 (http://frodo.wi.mit.edu/primer3/). Details of primer sequences and PCR conditions are available upon request. The PCR products were then sequenced in both directions on an ABI 3730 XL Automated DNA Sequencer with the ABI BigDye Terminator v3.1 cycle sequencing kit (Applied Biosystems). Sequencing of the *RNF213* gene was also performed in 20 unrelated healthy controls. The results were blasted and analyzed with the *RNF213* reference DNA sequence (NG_031980.1) utilizing Mutation Surveyor V4.0.6 software (SoftGenetics, State College, PA). Secondary structure of the RNF213 protein (UniProtKB_Q63HN8) was predicted by PredictProtein (https://www.predictprotein.org/).

**Table 6:** Association between RNF213 A4399T with clinical characteristics in MMD patients.

		Genotype frequency, no. (%)				Allele frequency, no. (%)			
SNP, population	No. of subjects	Minor homozygote	Heterozygote	Major homozygote	P value	Minor allele	Major allele	P value	OR (95%CI)
Genotype or allele		A/A	A/G	G/G		A	G		
Total cases	170	1	27	142		29	311		
Age group									
<18years	21	0	1	20	0.301	1	41	0.128	0.24(0.03–1.78)
≥18years	149	1	26	122		28	270		
Gender									
Female	74	0	13	61	0.599	13	135	0.883	1.06(0.49–2.28)
Male	96	1	14	81		16	176		
Symptoms at onset									
Hemorrhage	86	0	21	65	0.006	21	151	0.014	2.78(1.20–6.47)
Ischemia	84	1	6	77		8	160		
Symptoms at onset & ≥18 years (149)									
Hemorrhage	85	0	21	64	0.015	21	149	0.044	2.44(1.00–5.92)
Ischemia	64	1	5	58		7	121		

### Genotyping of RNF213 A4399T with unlabeled probe high resolution melting analysis

Genotyping of *RNF213* A4399T (rs148731719) was performed in all the 170 MMD patients and 507 controls with unlabeled probe high resolution melting. The nucleic acid sequences of the primers were designed as RNF213-4399F: 5′-aaaatttccccctcaaatgg-3′, RNF213-4399R: 5′-aattgtccacgagcgatgtt-3′ and the 3′-end blocked oligonucleotide probe was 5′-caatgtgaagctgtgagcaaat-3′. The PCR reaction and subsequent HRMA procedure was identical with the protocol described in the *Genotyping of RNF213 R4810K with unlabeled probe high resolution melting* section.

### Genotyping of *RNF213* P4007R, Q4367L, T4586P, L4631V, E4950D, A5021V and M5136I with high resolution melting analysis

Genotyping of the other *RNF213* variations identified through the genomic sequencing was performed in all the 170 MMD patients and 507 controls with high resolution melting analysis utilizing primers listed in Table S1. PCR was performed on a Rotor-Gene Q (Qiagen) in 20 μl of reaction volume. The master mix contained: 1×reaction buffer (TaKaRa BIO, Shiga, Japan), 1×SYTO9 (Invitrogen, Carlsbad, CA), 200 μM dNTP, 0.05 μM forward-primer, 0.5 μM reverse-primer, 1.5 mM MgCl_2_, 1 U TaKaRa Ex Taq DNA polymerase (TaKaRa BIO), and 1 μl (15–25 ng/μL) of genomic DNA. All amplifications began with a 5 minute hold at 95°C followed by 35 cycles of denaturation at 95°C for 30 seconds, annealing at 60°C for 30 seconds, and an extension at 72°C for 10 seconds.

The PCR products were subjected to denaturation at 98°C for 2 minutes followed by cooling down to 40°C for 2 minutes to facilitate the heteroduplex formation, and then heated slowly at 0.1°C/s from 75°C to 95°C. HRMA was performed using Rotor-Gene Q 1.7 software. Positive controls were included with each assay (homozygous wild-type and heterozygous variants).

### Statistical analysis

Differences in genotype and allele frequencies between patients and controls were compared. Differences in allele frequency was quantified by odds ratios (OR) and 95% CI. Chi-square distribution was used to analyze the associations between clinical characteristics and genotypes. *P* values under 0.05 were considered statistically significant. All the allele, genotype and linkage disequilibruium analyses were performed with the SHEsis software (http://analysis.bio-x.cn/myAnalysis.php).

## Results

### Establishment of the unlabeled probe HRMA

The *RNF213* R4810K mutation was detected by unlabeled probe HRMA with SYTO-9, the saturating HRM dye, using a RotorGene Q system. Figure 2 shows derivative melting curves in the probe detecting region. Three genotypes of R4810K (AA, AG, and GG) were accurately distinguished. A closer examination of the region of probe melting showed that samples with the A allele had a derivative melting peak at 66.4°C, and samples with the G allele showed a melting peak at 70.5°C The heterozygous samples showed two peaks, one at each temperature, representing the combination of both alleles (Figure 2a). All genotypes were clearly distinguished by analyzing the normalized melting unlabeled probe region. The difference curves generated by subtracting one heterozygote curve gave a much clearer view for genotype discrimination (Figure 2b). This method was used in the present study to screen samples. A single probe was able to recognize all three genotypes within the given sample set.

The genotype analysis by unlabeled probe HRMA completely matched the results obtained by sequencing for all detected samples.

### Association of *RNF213* R4810K with risk of MMD in Chinese population

Genotype distributions for *RNF213* R4810K mutation were in Hardy-Weinberg equilibrium in both MMD patients and normal controls. The genotype and allele frequencies of the mutation in MMD patients and healthy controls are shown in Table 2. R4810K mutation greatly increased the risk for MMD (OR = 36.7, 95% CI: 8.6∼156.6, *P* = 6.1 E-15), which is consistent with reports from Liu et al. (OR = 14.7, *P* = 10 E-4) [20]. The homozygous type (A/A) was not found among the normal controls. Four of the five patients with familial MMD had the *RNF213* R4810K mutation. They came from two different MMD families and one was the only A/A genotype found in this study, whose genetic backgrounds were demonstrated in Figure 3.

Between these two groups, differences in the distribution of both alleles and genotypes show statistical significance (Table 2). Based on the data previously identified by Kamada [19], our sample size would provide sufficient power (>80%) to identify a genetic association between the *RNF213* R4810K mutation and MMD.

### Association of *RNF213* R4810K with clinical characteristics in MMD patients

The allele frequency of *RNF213* R4810K was significantly different between the ischemia and hemorrhage groups (OR = 5.4, 95% CI: 1.8–16.1, *P* = 0.001) (Table 3). Ischemia was observed in every pediatric age patient, which may indicate statistical bias. We then observed the distribution of alleles in the adult group and found significant difference in genetic associations between the ischemia and hemorrhage groups (OR = 4.7, 95% CI: 1.5–14.8, *P* = 0.004) (Table 3). We found no association between genotype frequency and other characteristics of MMD.

### Identification of other *RNF213* variants

The lower mutation rate of R4810K in Chinese compared with Japanese and Korean indicate the possible existence of other variants in this gene. In the twenty disease cases subjected to sequencing, we found eight non-R4810K heterozygous variants in nine patients that were not present in the control group. Five previously identified variants [20] were detected. Among them, g.125960G>C (p.E4950D), g.120087A>C (p.T4586P), g.128375C>T (p.A5021V, rs138130613) and g.129275G>A (p.M5136I) were found in four different patients and g.115456G>A (p.A4399T, rs148731719) was also found in four MMD patients. Three novel variants, including g.107149C>G (p.P4007R), g.114926A>T (p.Q4367L) and g.120781C>G (p.L4631V) were identified in three different patients respectively. HRMA was employed to evaluate the distribution of all these variants except the highly frequent A4399T variation. HRMA identified a heterozygote of T4586P in the rest 150 MMD cases, but no more of these variants was found in both the 150 cases and control group. Secondary structure computer simulation suggested all these variations are involved in the helix, loop or sheet structure of this protein. Although the genetic variation pattern varies in different ethnic groups, the highly variable regions of this gene remain relatively intensive and conservative. None of these variants was found among the control group. The genetic and protein structure distribution of these variations are listed in Table 4. All the currently found MMD related variants were showed in Figure 1b and the newly identified variations were illustrated in Figure 4.

### Association of *RNF213* A4399T with risk and clinical subtyping of MMD in Chinese population

Because of the high frequency of *RNF213* A4399T (4/20) in MMD patients, this variant was further analyzed. Genotype distributions for *RNF213* A4399T mutation were in Hardy-Weinberg equilibrium in both MMD patients and normal controls. Table 5 illustrates the genotype and allele frequencies of the mutation in MMD patients and healthy controls. The A4399T variation, though found to be a common polymorphism in both case and control groups, also increased the risk for MMD (OR = 2.0, 95% CI: 1.2∼3.3, *P* = 0.004). Difference in the distribution of genotypes (*P* = 0.01) also showed statistical significance. The homozygous type (A/A) was not found among the normal controls. In the five patients from MMD families, a mother and her daughter were found to have the heterozygous A4399T polymorphism (Figure 3). We found no linkage disequilibrium (LD) between R4810K and A4399T (D' = 0.496, r^2^ = 0.00).

We found a significant difference in both the *RNF213* A4399T genotype (*P* = 0.006) and allele frequency (OR = 2.8, 95% CI: 1.2–6.5, *P* = 0.014) between the hemorrhage and ischemia group. Excluding all the pediatric patients, there is also a significant difference in the frequency distribution of genotype (*P* = 0.015) and alleles (OR = 2.4, 95% CI: 1.0∼5.9, *P* = 0.044) between the ischemia and hemorrhage groups (Table 6). No association was found between this genetic variation and other clinical characteristics.

## Discussion

*RNF213* encodes a protein-harboring RING (Really Interesting New Gene) finger motif that functions as an E3 ubiquitin ligase. *RNF213* knockdown zebrafish sprouted severely abnormal blood vessels in the head region, suggesting that *RNF213* is involved in a novel signaling pathway in intracranial angiogenesis [20]. There were three recent studies supporting the conclusion that RNF213 is related to MMD susceptibility [19]–[21]. Given the high incidence and prevalence of MMD in East Asia relative to the American and European populations, we developed an HRMA to detect an *RNF213* variant and conducted an association study between the genotypes and clinical parameters of MMD in a Chinese Han population. We found R4810K in 4 of 5 patients with familial MMD (80%), in 18 of 165 patients with nonfamilial MMD (10.9%), and in 2 of 507 normal control Chinese individuals (0.39%).

MMD is a rare sporadic disorder with a complex etiology [22]. However, highly variable incidence rates have been observed in different ethnic groups, and there are known familial occurrences (9–15% of all cases). Together, these factors make seem likely that this disease has a genetic cause or component [23]. The lack of systematic epidemiological investigations into MMD in China leaves us with a dearth of information regarding the annual incidence rates. However, the prevalence of reported cases of MMD has increased in recent years, partly due to increased access to noninvasive diagnostic tools, such as magnetic resonance angiography. Our study suggested that MMD was more common in male Chinese patients than in female Chinese patients (1.3∶1) and the incidence of MMD is higher in adults than in children (7.1∶1), indicating that clinical characteristics of the MMD in China is different from those in Japan, where the male to female ratio was 1∶1.8. The age at onset was under 10 years in 47.8% of Japanese patients [9].

Genome-wide association study has revealed *RNF213* to be the first MMD gene [19]. The subsequent large scale sequencing study from Liu et al. confirmed R4810K as the founder mutation in 42 MMD pedigrees [20]. Our results are consistent with those of the previous studies, showing the *RNF213* R4810K mutation to be significantly associated with MMD in Han Chinese.

A recent epidemiological survey in Nanjing, China revealed that the overall annual prevalence of MMD was 3.92/100,000 in east China, which was lower than the prevalence 10.5/100,000 found in Japan, but the results were similar with that of the reports from Taiwan [6], [24]. Epidemiological data show strong regional differences with a high occurrence in Asian countries (primarily Japan and Korea) and smaller rates in western countries, making the reported prevalence and incidence quite variable. MMD is extremely uncommon in non-Asian populations. Kamada et al. found the carrier frequency of R4810K in Japan to be 1/72 [19]. In contrast, no R4810K carrier was found in 400 Caucasian controls. In our study, the carrier frequency of R4810K in Chinese was 2/507, much lower than that of the Japanese cohort, in agreement with the recent finding of distribution of R4810K in Eastern and Southeastern asian population [25]. These results suggest that the genetic background of MMD in the Chinese populations is distinct from that in the Japanese and Western ones and the low incidence of MMD in Chinese populations may be attributable to a low frequency of the founder *RNF213* mutation.

The clinical manifestations of MMD consist of intracerebral hemorrhage, cerebral ischemia (TIAs and cerebral infarction), and other symptoms such as headache and seizures [26]. A bimodal age distribution of MMD has been reported in all children (<18) presenting ischemic symptoms and adults (≥18) presenting hemorrhage (59%, 53 of 90) [27], [28]. The potential role of mutation in MMD was hypothesized because of its association with some of the clinical symptoms (e.g., hemorrhage or ischemia) of MMD. The frequency of the mutant A allele of *RNF213* R4810K was higher in the group with ischemia, than in the hemorrhage group. Because the initial symptom of all patients in pediatric group is cerebral ischemia in our study, the distribution frequency of the mutation was analyzed in the adult group. The frequency of the mutant A allele was significantly higher in the ischemia group than in the hemorrhage group, suggesting that clinical manifestations of MMD may be associated with genetic background. This finding is consistent with recent research demonstrating that infarctions at initial presentation were of significantly higher frequency in homozygotes than in heterozygotes and wild types of R4810K [21]. Our finding may partly explain why these manifestations vary by geographic region and ethnic group.

A previous study revealed additional novel variants only in Chinese cohort, but not in Japanese and Korean cases, highlighting genetic heterogeneity of MMD among the three populations in East Asia (20). Therefore, we sought to investigate other non-R4810K variants in MMD. Firstly, we built up a genetic variation heat map of *RNF213* with the 1000 genomes database and located the high variable region of this gene (from exon 40 to exon 68). After direct sequencing of this region in both 20 cases and 20 controls and melting analysis validation, did we identify seven rare non-R4810K heterozygous variants present in the 170 MMD cases and not in the 507 controls. Two of them, i.e. E4950D and A5021V were found in two different patients and recently reported by Liu et al [20]. Another two variations, T4586P and M5136I were listed in the 1000 genome project database and found in two and one MMD cases respectively. The other three novel variations, P4007R, Q4367L and L4631V were also identified in three different MMD patients respectively. Since the frequency of these variants was low, we surmise these seven rare variants are incidental variations of MMD. Further studies in protein function and structure of RNF213 would probably help to define if these variants are the true pathogenic MMD mutations.

Due to the high prevalence of A4399T variation (20%) in the 20 patients subjected to sequencing, we then performed an unlabeled probe HRMA in all the cases and controls. The results suggested the A4399T variation to be a potential risk factor for MMD, especially the hemorrhage subtype. The frequency of the minor allele among the present 507 Chinese subjects was 0.044, which is in agreement with that reported for Asian subjects in 1000 genomes project. This variation is therefore more common in Asian compared with other ethnicities (EUR 0.009; AMR 0.008; AFR 0.000), and this variant could partly explain the difference of prevalence of MMD between Asian and non-Asian populations.

Compared with R4810K, A4399T is less presented in the familial cases (4/5 versus 2/5), further confirmed the R4810K mutation as a main genetic driver variant for MMD. Furthermore, different variations with no LD (R4810K and A4399T) were involved in pathogenic risks of different clinical manifestations (ischemia and hemorrhage). These noticeable phenomena incontrovertibly indicated structure alterations in *RNF213* could be potential disease drivers of MMD.

This study has several limitations. First, it involved a relatively small number of patients. Larger studies are needed to verify our observations. Second, since we only applied gene scanning method at the genetic level, we cannot provide evidence regarding that the variant RNF213 is connected to any impairment of physiological function in MMD. In the future, more extensive studies involving structure biology will be conducted to rule out the function changes in protein induced the genetic variations.

We believe differences in the genetic background leads to the distinct mutation patterns among different ethnic groups. This study adds novel *RNF213* mutations to those previously reported, providing further evidence for genetic and phenotypic heterogeneity in MMD. Taken together, these results not only suggest that the *RNF213* gene is associated with MMD in the Chinese Han population but also imply that the mutational spectrum of *RNF213* gene is different from that found in other populations.

## Supporting Information

Table S1 **Primer sequence used in high resolution melting genotyping of P4007R, Q4367L, T4586P, L4631V, E4950D, A5021V and M5136I.**
(DOC)
