# Supplementary material for: Molecular Analysis of RNF213 Gene for Moyamoya Disease in the Chinese Han Population
Source: PLoS One. 2012 Oct 23;7(10):e48179. doi: 10.1371/journal.pone.0048179 (PMC3479116; doi:10.1371/journal.pone.0048179)
Supplement: Table S1 — Primer sequence used in high resolution melting genotyping of P4007R, Q4367L, T4586P, L4631V, E4950D, A5021V and M5136I. (DOC) [file pone.0048179.s001.doc]

**Table S1 Primer sequence used in high resolution melting genotyping of** P4007R, Q4367L, T4586P, L4631V, E4950D, A5021V and M5136I

| Primer | Seuqence | Amplicon size (bp) |
| --- | --- | --- |
| 4007-F | ATTCAGCCGTGCTCCATCT | 112 |
| 4007-R | TCATCTGCTCTGAGGCAAAC |  |
| 4367-F | TTGATACCCTTGATTTTGCAG | 114 |
| 4367-R | GCTTGCATTGTGGGATCTGT |  |
| 4586-F | CCCAGTGGTCTTCCTCCTTA | 95 |
| 4586-R | GTTTGGCAGCACTGGGTTAT |  |
| 4631-F | CTCCAGTGAGGGATCCAAAA | 146 |
| 4631-R | CTGGTGCTGCTCTTGGAGA |  |
| 4950-F | ACTGCCAGTACCAGGTGGAG | 73 |
| 4950-R | ATCTGCCGCTGAATCTTCTC |  |
| 5021-F | TAGTGCCATCAGTGGACAGC | 85 |
| 5021-R | GCTCAGAAACCCCAGAGTGA |  |
| 5136-F | CTAGACGCCTTCCTGCTAGA | 109 |
| 5136-R | GTGCAAATCCATACCTCCA |  |
